# Publishers’ Notice

**Published:** 1869-10

**Authors:** 


### *PUBLISHERS' NOTICE.*

FROM the date of the present number, the undersigned will assume the publication of the CHICAGO MEDICAL JOURNAL, and the exclusive management of its financial and business affairs.

In undertaking this enterprise they are actuated by a desire to extend their relations with the profession with which they have been closely identified many years as the purveyors of medical literature in the Northwest. Believing that this intimate association, by familiarizing them with the literary wants of the medical profession, has given them peculiar and unusual facilities for the satisfactory prosecution of such work, they confidently ask the countenance and co-operation of old friends who have sustained the JOURNAL during the twenty-six years past, both by continued subscription and frequent communication of the important results of their experience and observation to its pages.

The typographical and general mechanical excellence of successive numbers will be brought up to an equality with the best magazines of the country, and especial attention will be paid to its prompt issue at or before the monthly dates. The monthly form will be resumed, it having been found by experience that this gives better opportunity for a variety of matter, without detracting from space for admission of elaborate essays.

Specimen copies will be furnished without charge. To all new subscribers remitting subscription price, $3, for 1870, the remaining numbers of the present year will be sent free. Address,

W. B. KEEN & COOKE,
113 *and* 115 *State Street, Chicago.*

